# Yield of Systematic Longitudinal Screening of Household Contacts of Pre-Extensively Drug Resistant (PreXDR) and Extensively Drug Resistant (XDR) Tuberculosis Patients in Mumbai, India

**DOI:** 10.3390/tropicalmed5020083

**Published:** 2020-05-26

**Authors:** Roma Haresh Paryani, Vivek Gupta, Pramila Singh, Madhur Verma, Sabira Sheikh, Reeta Yadav, Homa Mansoor, Stobdan Kalon, Sriram Selvaraju, Mrinalini Das, Chinmay Laxmeshwar, Gabriella Ferlazzo, Petros Isaakidis

**Affiliations:** 1 Médecins Sans Frontières (MSF)/Doctors Without Borders, Mumbai 400088, India; MSFOCB-Mumbai-OS@brussels.msf.org (R.H.P.); msfocb-mumbai2@brussels.msf.org (P.S.); sabirayasin74@gmail.com (S.S.); reeta242015@gmail.com (R.Y.); msfocb-delhi-med@brussels.msf.org (H.M.); Msfocb-India-ops-strategic-advisor@brussels.msf.org (S.K.); chinmay2210@gmail.com (C.L.); 2 Dr RP Centre for Ophthalmic Sciences, All India Institute of Medical Sciences (AIIMS), New Delhi 110029, India; vgupta@aiims.edu; 3 Department of Community & Family Medicine, All India Institute of Medical Sciences (AIIMS), Bathinda, Punjab 151001, India; drmadhurverma@gmail.com; 4 National Institute for Research in Tuberculosis, Chennai 600031, India; sriram.s@nirt.res.in; 5 Southern Africa Medical Unit (SAMU), Médecins Sans Frontières, Cape Town 7925, South Africa; Gabriella.FERLAZZO@joburg.msf.org (G.F.); Petros.Isaakidis@joburg.msf.org (P.I.)

**Keywords:** incidence, tuberculosis, household contacts tracing, operational research

## Abstract

While risk of tuberculosis (TB) is high among household contacts (HHCs) of pre-extensively drug resistant (pre-XDR) TB and XDR-TB, data on yield of systematic longitudinal screening are lacking. We aim to describe the yield of systematic longitudinal TB contact tracing among HHCs of patients with pre-XDR-TB and XDR-TB. At the Médecins Sans Frontières (MSF) clinic, Mumbai, India a cohort comprising 518 HHCs of 109 pre-XDR and XDR index cases was enrolled between January 2016 and June 2018. Regular HHC follow-ups were done till one year post treatment of index cases. Of 518 HHCs, 23 had TB (21 on TB treatment and two newly diagnosed) at the time of first visit. Of the rest, 19% HHCs had no follow-ups. Fourteen (3.5%) TB cases were identified among 400 HHCs; incidence rate: 2072/100,000 person-years (95% CI: 1227–3499). The overall yield of household contact tracing was 3% (16/518). Of 14 who were diagnosed with TB during follow-up, six had drug susceptible TB (DSTB); six had pre-XDR-TB and one had XDR-TB. Five of fourteen cases had resistance patterns concordant with their index case. In view of the high incidence of TB among HHCs of pre-XDR and XDR-TB cases, follow-up of HHCs for at least the duration of index cases’ treatment should be considered.

## 1. Introduction 

Tuberculosis (TB) is a long-standing global health issue, disproportionately affecting low- and middle-income countries. India is a country with a high TB burden [1]. Drug-resistant tuberculosis (DR-TB) is also reported to have an increasing trend in the country. Pre-extensively drug resistant (pre-XDR-TB) is the presence of resistance to rifampicin and isoniazid along with resistance to either one of the fluoroquinolones (ofloxacin, levofloxacin or moxifloxacin) or second-line injectables (amikacin, capreomycin or kanamycin). In case the TB bacilli have resistance to both fluoroquinolones and second-line injectables, along with rifampicin and isoniazid, they are classified as extensively drug resistant (XDR). In India, the estimated incidence of TB in India was 2,700,000 [2]. According to the drug-resistance survey report, in India, 28% patients with TB have resistance to any anti-tubercular drugs and 6.2% have multi-drug resistant TB (MDR-TB); among MDR-TB, 21.8% have pre-XDR-TB and 1.3% have XDR-TB [3]. Some studies from the country have reported a higher prevalence of XDR-TB [4,5,6].

Systematic reviews suggest that contact investigations are an important intervention in early identification of active TB cases, thus minimizing TB transmission [7,8,9]. Fox and colleagues have reported active TB among 3.4% contacts of patients with multi-drug-resistant or extensively drug-resistant TB in low-and middle-income settings [9]. In Pakistan, baseline contact investigation of index cases with MDR-TB identified MDR-TB in 17.4% and DS-TB in 4.2% of close contacts [10]. A longitudinal study of household contact (HHC) among newly diagnosed TB patients in Vietnam reported incident TB in 0.5% of contacts at 12-month follow-up and in 0.3% of contacts at 24-month follow-up, while the prevalence at baseline evaluation was 0.5% [11]. In China, four-year follow-up of close contacts of bacteria-positive TB index cases reported a high risk of incident TB (333/100,000 person years) [12].

To add to the evidence around longitudinal contact tracing in addition to baseline contact tracing among drug-resistant TB cases, the present study aimed to determine the yield of systematic longitudinal household contact tracing among patients with pre-XDR-TB and XDR-TB initiated on treatment at Médecins Sans Frontières (MSF) Clinic, Mumbai. Secondary objectives included (1) assessment of profile of index cases and HHCs developing TB and (2) drug resistance patterns in TB diagnosed among HHCs.

## 2. Materials and Methods

### 2.1. Study Design

This was a retrospective cohort study using routine program data.

### 2.2. Setting

The study was done in Mumbai, which is among the most populous cities of India. Located in the western part of India in the state of Maharashtra, it has a population density of 21,000/km^2^. The city has high rates of migration and is home to a large slum population. Nearly 9895 patients with DR-TB were diagnosed in Maharashtra, and TB case notifications were nearly 209,642 in 2018 [2,12,13,14].

The MSF clinic in Mumbai started offering treatment to patients with pre-XDR and XDR-TB in 2012 [15]. Eligibility criteria for enrollment are based on resistance profile and treatment history. Individualized treatment is offered on an outpatient basis based on drug sensitivity patterns. Bedaquiline and Delamanid are provided to patients who require it. All patients receive free-of-charge services, which include counseling, diagnosis, treatment, social enabler (dry ration), travel support and medical reimbursement for co-morbid conditions as per need. Treatment is provided using an ambulatory model of care described elsewhere [15].

#### 2.2.1. Household Contact (HHC) Investigation

Since 2013, the clinic has been performing investigation of HHCs of registered pre-XDR and XDR-TB patients. HHCs of DR-TB cases were members of the household regularly living with the patients registered for care in the MSF Clinic (hereafter mentioned as index cases). At enrollment of a new index case, a trained nurse visited their homes, a list of HHCs was prepared, and details were noted in a standardized form. All the HHCs are examined for signs and symptoms of TB and following risk factors: pregnancy, diabetes mellitus, HIV, alcohol use disorder, drug use, previous history of TB, age >55 years and immune-suppressive therapy.

Adults and adolescents HHCs over 15 years of age with risk factors and all pediatric (aged <15 years) HHCs are invited to visit the MSF clinic for a detailed screening. Those who visit the clinic are assessed using chest X-ray (CXR) and physical examination. If they have symptoms suggestive of TB or CXR abnormality, sputum examination or gastric lavage for smear, cartridge based nucleic acid amplification test (CBNAAT)/GeneXpert and culture and drug susceptibility testing (C-DST) are done. HHCs that are diagnosed with active TB are initiated on treatment as per their TB drug susceptibility pattern.

#### 2.2.2. Follow-Up of HHCs

HHCs aged >15 years and not having any high-risk factors are followed every six months in their homes. Those who have risk factors are followed at three-monthly intervals during the first year and then six-monthly at home. All children <15 years are followed up three-monthly at the MSF clinic. At each follow-up visit, screening of contacts is done for presumptive TB, defined as any of the symptoms/signs: (a) cough >2 weeks, (b) fever >2 weeks, (c) significant weight loss, (d) any abnormality in chest radiograph. Contacts with presumptive TB undergo detailed clinical, radiological and laboratory evaluations. All laboratory investigations are done in an accredited private laboratory contracted by MSF. In case a contact does not report for follow-up at the clinic, they are contacted telephonically and encouraged to visit the clinic. The follow-up continues for the entire duration of TB treatment of the index case and one year after treatment completion. In case of the death of the index case, follow-up of HHCs is still offered for the next one year.

### 2.3. Study Population and Participants

For the present analysis, we included all HHCs of index cases with pre-XDR and XDR-TB registered for treatment between January 2016 and June 2018. Index cases who did not have bacteriologically confirmed pre-XDR or XDR-TB, and those who refused treatment, were excluded.

### 2.4. Data Variables and Sources, Data Analysis

Data for each index case and HHCs is maintained in EMR software (Bahmni^™^), individual care record files, and in Microsoft Excel^™^ spreadsheets. Data for this study were extracted from these sources. Data was analyzed using EpiData Analysis (version 2.2.2.178 EpiData Association, Odense, Denmark) and Stata version 15.1 (StataCorp, College Station, TX). The numbers of HHCs assessed at each stage of follow-up were noted to understand the contact investigation cascade. Survival analysis was used to analyze the incidence rate of incident TB overall, by age, sex and time from follow-up initiation. HHCs were censored in case of loss to follow-up (LTFU), completion of follow-up period, or on 30^th^ April 2019, whichever was earlier. The date of last visit was taken as the date of censoring. Per-capita floor area of the house was calculated, and overcrowding was considered present in case the per-capita area was less than 50 square feet. In all analyses, p < 0.05 was considered statistically significant. Wherever appropriate, 95% confidence intervals (95% CI) are being reported.

### 2.5. Operational Definitions

(1) Index case: patients with pre-XDR or XDR-TB registered for treatment in MSF Clinic.

(2) Incident TB: any HHC diagnosed as suffering from TB after one month of baseline contact evaluation.

(3) Lost to follow-up (LTFU): HHCs not evaluated during a scheduled visit and any time during the subsequent 6-month period.

### 2.6. Ethics

Ethics approval was obtained from the Ethics Advisory Group of the International Union Against Tuberculosis and Lung Disease, Paris, France (EAG No. 96/18). The study met the criteria for a posteriori analysis of routinely collected clinical data and did not require MSF Ethics Review Board full review. It was conducted with permission of the medical director, Operational Centre Brussels, MSF.

## 3. Results

**Figure 1:**
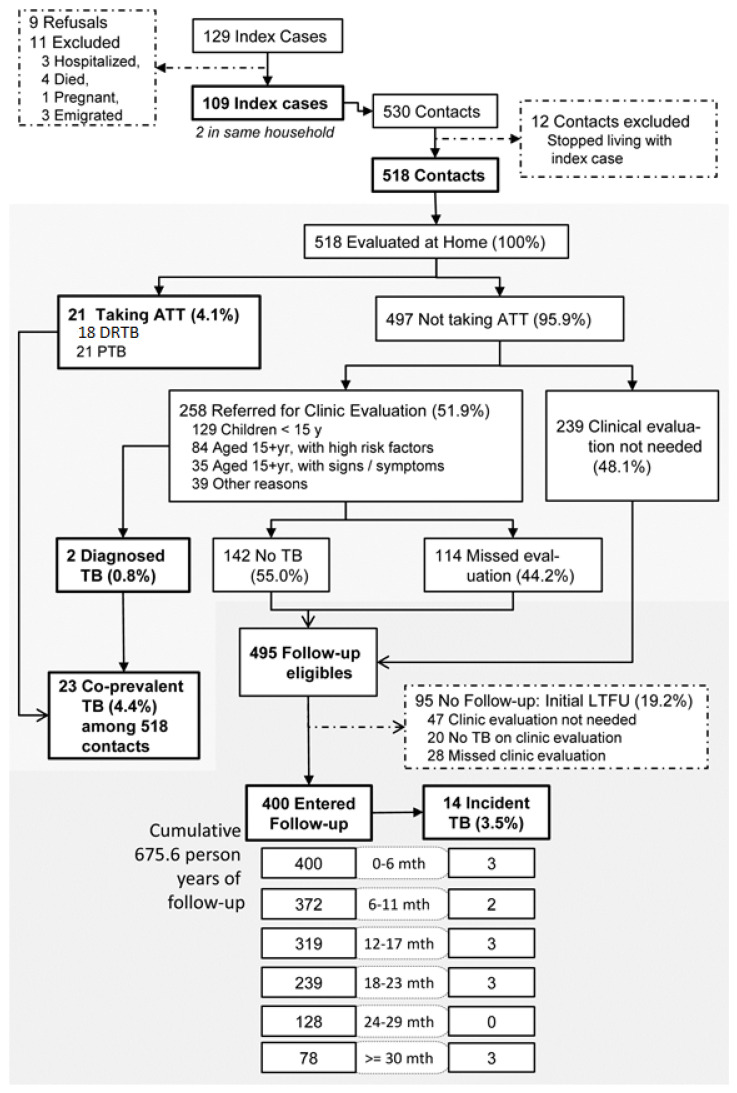
Assembly and follow-up of cohort comprising household contacts of index cases with pre-XDR and XDR tuberculosis registered at the MSF Clinic, Mumbai between Jan 2016 and June 2018. TB = Tuberculosis; DSTB = Drug-sensitive TB; DRTB = Drug-resistant TB; pre-XDR = Pre-extensively drug-resistant; XDR-TB = Extensively drug-resistant tuberculosis; PTB = Pulmonary TB; ATT = Anti-tubercular therapy; LTFU = Loss to follow-up. While 114 HHCs missed clinic evaluation, we considered them as eligible for follow-up since they were at risk of incident tuberculosis.

The clinic enrolled 129 cases of pre-XDR and XDR-TB during the study period; among these, we excluded 11, and nine refused treatment later, yielding 109 eligible index cases (Figure 1). There were 58 (53.4%) cases with XDR-TB; nine (8.3%) were under 15 years of age, and 60 (55.1%) were female (Table 1). Two index cases were from the same household. These 109 index cases had 530 HHCs, among whom 518 (97.7%) underwent baseline evaluation. Among 518 HHCs, 23 were reported to have TB at the time of first visit (21 already on treatment, two newly diagnosed). The rest of the 495 HHCs were eligible for follow-up.

### 3.1. TB Diagnosis among HHCs

Among the 495 HHCs eligible for follow-up, 95 (19.2%) did not complete the first follow-up visit (at 3 or 6 months, based on age and risk profile) and were early LTFUs. For the remaining 400 HHCs, median follow-up of 18.9 months [Interquartile range (IQR): 14.0–25.8] and cumulative follow-up of 675 person-years (py) was achieved. During the follow-up period, we identified 14 (3.5%) new TB cases (Figure 1). The median time to TB diagnosis among HHCs was 18 months (IQR: 6–21 months). The incidence rate of TB was 2072 (95% CI: 1227–3499) per 100,000 person-years over the entire follow-up period.

Of 14 HHCs with incident TB, ten (69.2%) incident cases were 15 years of age or older, and eight (61.5%) were female (Table 2). Six cases occurred in siblings of index cases and three in children. None of the index cases or HHC characteristics were associated with incident disease in univariate analysis.

### 3.2. Overall Yield of TB in HHCs

The overall yield among 518 identified HHCs was 3%. A total of 16 patients were diagnosed with TB, which included two patients who were diagnosed with TB on first day of screening and 14 who were identified during follow-up.

### 3.3. Drug Resistance Patterns

Among 37 identified TB cases among HHCs, the TB resistance profiles of 23 patients who were reported with TB on the first day of screening (including 21 on TB treatment and two newly diagnosed) were as follows: three had DSTB, seven had MDR TB, five had pre-XDR, and eight had XDR-TB, respectively. Among 14 cases found during follow-up, six had DSTB, six had pre-XDR-TB, one had XDR-TB and a TB resistance profile could not be ascertained for one. Five (38.5%) of these 14 cases were concordant with index case (4 Pre-XDR-TB and 1 XDR-TB).

## 4. Discussion

The overall yield was 3% during the longitudinal household contact tracing for TB in patients diagnosed with pre-XDR and XDR-TB receiving care in MSF Clinic in Mumbai, India. Through systematic longitudinal evaluation of HHCs, the study reported a higher incidence of TB disease than the previously reported study from India [2].

The yield of TB reported in our study is comparable to findings of systematic reviews [6,7,8]. However, the systematic review by Morrison et al. [6] also included studies where TB index cases were bacteriologically and clinically confirmed patients, while our study included only biologically confirmed cases as index cases.

Our findings are in line with those of Leung and colleagues, who conducted follow-up of HHCs of MDR-TB and reported that presence of XDR-TB significantly increased the odds of identifying a prevalent TB as well as hazard of incident case by almost five times after 18 months [16]. However, they did not have pre-XDR cases in their cohort. The observed incidence is higher than in China, Gambia and Peru [17,18,19], though it matches results from a Vietnam active case finding setting [10].

We observed discordant TB resistance profiled in HHCs compared to index cases; about half of the cases were DSTB, while the index cases were either pre-XDR-TB or XDR-TB [7]. These may hint towards community transmission of DSTB in a high burden setting.

### 4.1. Limitations

The study could not differentiate whether incident cases were the result of infection within the household or community acquired, since we did not conduct molecular fingerprinting. Molecular fingerprinting would have assisted in identifying the transmission pathways. We also did not evaluate for latent TB infection. Given the high incidence of TB in the program setting, it is likely that transmission would have occurred outside households, as is suggested by recent literature [20]. We observed that 44.2% of HHCs did not come for baseline clinic evaluation and 19.2% of HHCs had initial LTFU. A commonly reported reason for this was family members moving away from the index case. The index cases were largely slum-dwellers staying in rented accommodation, and the family members moving away could be a result of stigma, or a reaction to protect the health of family members. Finally, our findings may be representative of an urban Mumbai slum context and not directly applicable in other settings.

### 4.2. Implications for Policy and Practice

Under the Programmatic Management of Drug Resistant TB (PMDT) in India, it has been recommended that all close contacts of DR-TB cases be identified through contact tracing and evaluated for active TB disease. If the contact is found to be suffering from TB disease, irrespective of bacteriological confirmation, s/he should be identified as “presumptive DR-TB” and therapy be initiated based on their history of previous anti-TB treatment or TB resistance profile of index TB case. Simultaneously, two sputum samples should be transported for culture and DST. No definitive guidelines have been provided for prophylaxis among contacts with no active disease, and follow-up duration and close monitoring is suggested as the recommended action [21]. Our results suggest the need for follow-up of HHCs of patients with DR-TB after baseline evaluation for at least the duration of the index case’s treatment.

### 4.3. Implications for Future Research

Cost-effectiveness analyses have shown the utility of active case finding among HHCs [22]. Since we are proposing a longitudinal follow-up of contacts of pre-XDR and XDR cases, future research should focus on establishing the cost effectiveness of this approach. An assessment of latent TB infection among contacts of pre-XDR and XDR cases is also recommended in view of the high incidence rates of active infection. Molecular fingerprinting and linkage analyses will clarify the epidemiology of transmission. Longer follow-up of more pre-XDR and XDR contacts is also recommended in research settings along with analyses of implementation effects of various screening preventive and infection control strategies so that their relationship with incident disease may be better understood.

## 5. Conclusions 

We observed a high incidence of TB among HHCs of pre-XDR and XDR-TB in Mumbai, India. Systematic longitudinal TB screening of HHCs in cases with pre-XDR and XDR-TB must be strengthened, as they are at a high risk of incident disease.

## Figures and Tables

**Table 1:** Characteristics of index cases with pre-XDR and XDR tuberculosis registered at the MSF clinic, Mumbai, India, January 2016 to June 2018 (N = 109). IQR: Inter-quartile range.

Characteristics	Groups	Index Cases	
		N	%
Type of TB drug resistance	Pre-Extensive Drug Resistance (pre-XDR)	51	(47)
	Extensive Drug Resistance (XDR)	58	(53)
Age at enrolment (years)	0–14	9	(8)
	15 and above	100	(92)
Sex	Male	49	(45)
	Female	60	(55)
Previous TB treatment	No treatment	6	(6)
	History of previous TB treatment	103	(94)
Site of TB in Index Case	Extra-pulmonary (EPTB)	15	(14)
	Pulmonary (PTB)	94	(86)
X-ray results (n = 105)	Abnormal	92	(88)
	Normal	13	(12)
Sputum conversion (in months) [Median (IQR)] (n = 76)		2 (1–4)	
Human Immunodeficiency Virus (HIV) co-infection		5	(5)
Diabetes mellitus co-morbidity		10	(9)
Overcrowding (area per person <= 50 square feet)		60	(55)
Staying in rented house		33	(30)

**Table 2:** Profile and factors associated with incident TB among household contacts of patients with pre-XDR and XDR tuberculosis in Mumbai, India, January 2016–June 2018 (N = 400). * Column percentage; ** Row percentage; N(%) were compared using the Chi-square test or Fisher’s exact test; †: Median (Inter-quartile range), compared using the Wilcoxon rank-sum test; Pre-XDR TB: Pre-extensive drug resistant TB; XDR-TB: Extensive drug-resistant TB; PTB = Pulmonary TB; EPTB = Extra-Pulmonary Tuberculosis; AFB = Acid fast bacilli.

Variable		Household Contacts (n = 400) *	Household Contacts with Incident TB(n = 14) **	*p*-Value
Index Case Characteristics		n (%)	n (%)	
Type of index case	Pre-XDR	166 (41)	7 (4)	0.51
	XDR	234 (59)	7 (3)	
Age (Median(IQR), years) ^†^		24 (19–30)	24 (18–26)	0.43
Sex	Male	156 (39)	6 (4)	0.76
	Female	244 (61)	8 (3)	
Prior TB treatment	No treatment	23 (6)	2 (9)	0.43
	TB treatment	377 (94)	12 (3)	
HIV in index case	No	384 (96)	12 (3)	0.10
	Yes	16 (4)	2 (13)	
AFB result	Negative	183 (47)	8 (4)	0.59
	Scanty	42 (11)	2 (5)	
	Positive	162 (42)	4 (3)	
Site of TB	EPTB	51 (13)	0 (0)	
	PTB	349 (87)	14 (4)	
X-ray results	Abnormal	344 (88)	14 (4)	
	Normal	47 (12)	0 (0)	
Number of household contacts ^†^		6 (4–8)	6 (5–10)	0.21
**Household Contact Characteristics**				
Age group	0–14 years	94 (23)	4 (4)	0.75
	15 years or more	306 (97)	10 (3)	
Sex	Male	205 (51)	6 (3)	0.52
	Female	195 (49)	8 (4)	
Relationship with index case	Child	68 (17)	3 (4)	0.37
	Parent	120 (30)	4 (3)	
	Sibling	97 (24)	6 (6)	
	Spouse	22 (6)	0 (0)	
	Other	93 (23)	1 (1)	
Area < 50 sq/feet/capita	No	226 (59)	10 (4)	0.32
	Yes	159 (41)	4 (3)	
Past history of TB	No	358 (90)	11 (3)	0.17
	Yes	42 (10)	3 (7)	
